# Bacteriome-based oral dysbiosis index in patients with oral squamous cell carcinoma

**DOI:** 10.1080/20002297.2026.2668149

**Published:** 2026-05-07

**Authors:** David Szaraz, Jan Bohm, Ctirad Machacek, Gabriela Salokova, Daniela Gachova, Filip Ruzicka, Zdenek Danek, Tarik Gheit, Jiri Zavadil, Petra Borilova Linhartova

**Affiliations:** a Clinic of Maxillofacial Surgery, University Hospital Brno, Brno, Czech Republic; b Faculty of Medicine, Masaryk University, Brno, Czech Republic; c RECETOX, Faculty of Science, Masaryk University, Brno, Czech Republic; d Department of Pathology, University Hospital Brno, Brno, Czech Republic; e Department of Microbiology, St. Anne’s University Hospital, Brno, Czech Republic; f Epigenomics and Mechanisms Branch, International Agency for Research on Cancer, Lyon, France

**Keywords:** Mouth neoplasms, microbiota, *Fusobacterium*, dysbiosis, *Candida*, cytomegalovirus, EBV, metagenomics, dental plaque, tongue

## Abstract

**Background:**

Oral dysbiosis plays an important role in the pathogenesis of oral squamous cell carcinoma (OSCC). Our study aimed to perform a pairwise comparison of the oral microbiota, especially the bacteriome, from OSCC tumoral surface vs other oral samples and evaluate the association of a novel bacteriome-based Oral Dysbiosis Index (bbODI) with the OSCC surface.

**Materials and methods:**

This pilot observational study used 84 patient-matched samples from the OSCC tumoral surface (swabs and biopsies), healthy oral mucosa (tongue and buccal swabs), and supragingival dental plaque swabs. Bacteriomes were analyzed by 16S rRNA amplicon sequencing. The presence of microscopic fungi and selected viruses was also evaluated.

**Results:**

The relative abundance of the genus *Fusobacterium*, the ratio of the relative abundances of gram-negative to gram-positive bacterial genera, and the bbODI on the tumour surface significantly differed from patient-matched healthy oral mucosa (both buccal and tongue swabs) and supragingival dental plaque samples. Oral candidosis was found in 25% of patients; all patients were negative for cytomegalovirus and Epstein–Barr virus.

**Conclusions:**

Certain characteristics of the bacteriome composition of the OSCC surface differ from patient-matched samples of healthy oral mucosa and supragingival dental plaque. The proposed bbODI appears to be a promising non-invasive tool for the identification of bacteriome disruption on the OSCC surface.

## Key messages

- A novel index (bbODI) was introduced based on the ratio of selected bacteria.
- The bbODI provides a comprehensive single-value characteristic of the oral bacteriome.
- The bbODI was significantly higher in swabs from OSCC than from healthy oral mucosa.

## Graphical abstract

**Figure:**
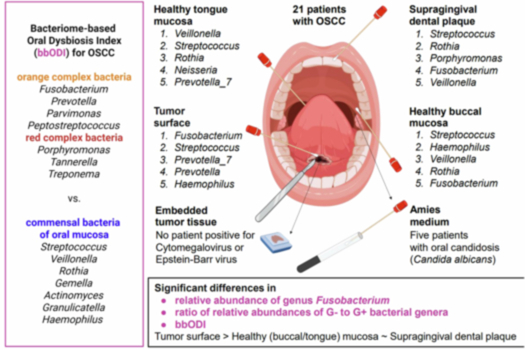

## Introduction

Oral squamous cell carcinoma (OSCC), a subgroup of head and neck squamous cell carcinomas (HNSCC), is characterized by relatively high incidence and low survival rates [1]. This is largely due to detection at late stages, despite the good accessibility of the oral cavity for direct examination. Tobacco smoking, alcohol drinking, and poor oral hygiene are considered major risk factors of OSCC [1]. All these exposure factors have also been reported to be associated with a shift in the oral microbiome profile [2,3].

Disruption of the balance in the oral microbiota can lead to oral dysbiosis, characterized by an increase in the relative abundance of gram-negative bacterial genera and yeasts associated with OSCC [4]. Several Oral Dysbiosis Indices (ODIs) have been recently suggested as valuable tools for understanding the complex microbial ecosystems of the oral cavity and their relationships with health and disease [5–10]; so far, however, no ODI based on bacteriome sampled preoperatively from the tumour site has been proposed and investigated from the perspective of their associations with OSCC.

The role of the oral microbiota and its products in OSCC pathogenesis has been intensively studied over the last decade [11,12]. The mechanisms by which the oral microbiota contributes to tumour growth and development include modulation of the balance between host cell proliferation and apoptosis, production of oncometabolites, activation of inflammatory processes, and promotion of tumour angiogenesis and tumour invasiveness [12]. Changes in the relative abundances of several oral bacterial species, such as orange/red complex periopathogens [13] (including *Fusobacterium nucleatum*) and microscopic fungi (especially *Candida* sp.), were already associated with OSCC [4,14]. Unlike for other HNSCC subsites, however, no links between the most common oncogenic viruses (human papillomavirus, HPV; Epstein–Barr virus, EBV; cytomegalovirus, CMV) and OSCC have been confirmed [4,15].

OSCC can occur in various areas of the oral cavity, such as the lateral/ventral tongue, floor of the mouth, and buccal mucosa [16,17]– sites differing in their microbial patterns even on healthy oral mucosa. The tongue, with its papillae, supports a more diverse microbial community, including anaerobes, while the buccal mucosa and the floor of the mouth typically harbour a less diverse, thinner biofilm [18]. Moreover, it is important to note that the presence of hard tissues in the oral cavity plays a crucial role in the overall oral microbiome. Poor oral hygiene and plaque accumulation in dentition predispose patients to oral dysbiosis [19]; changes in the composition and diversity of the dental plaque have been observed during OSCC progression [20]. Despite these well-established facts, no paired comparison of the microbiomes of the tumour surface, healthy oral mucosa, and dental plaque that would promote a comprehensive understanding of the microbial ecology in the oral environment in patients with OSCC has been conducted so far.

We hypothesized that the bacteriome characteristics of oral swabs differ according to the anatomical subsite in patients with OSCC, with anaerobic gram-negative genera being more abundant on the tumour surface than in those from patient-matched healthy mucosa. We also hypothesized that the bacteriomes of supragingival dental plaque samples would be more similar to those collected from the OSCC surface than to those collected from healthy buccal and tongue swabs. Specifically, this pilot study aimed (i) to describe and compare the oral bacteriomes of four different sites within the oral cavity of patients with OSCC, whose oral samples were also examined for microscopic fungi and selected oncogenic viruses. Further, we aimed (ii) to compare the oral bacteriomes on the tumour surface with those on patient-matched (i.e. tumour site-respecting) healthy mucosa, and (iii) to investigate the possible association of a newly proposed bacteriome-based ODI (bbODI) with OSCC.

## Materials and methods

### Study design

**Figure 1.**
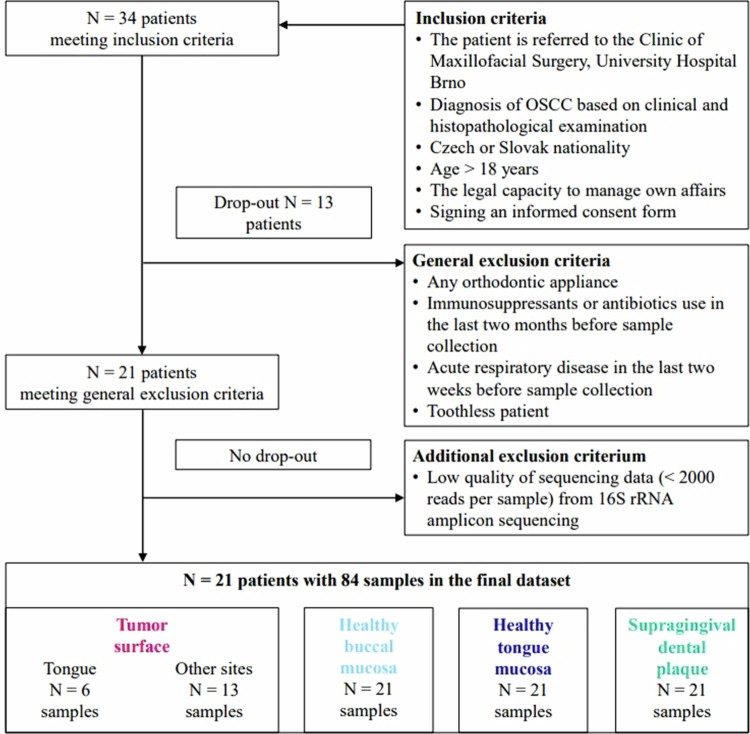
A flowchart of patient recruitment. *N*, number of patients; OSCC, oral squamous cell carcinoma.

The present pilot study was designed as a retrospective observational study focusing on bacteriome analysis in subject-matched oral swabs from patients with OSCC. Informed consent was obtained from all patients in line with the Helsinki declaration, prior to their inclusion in the study. The study was approved by the Ethics Committee of the University Hospital Brno (03-120220/EK; 2/2/2020). Patients with a histopathological diagnosis of OSCC were recruited from the pool of patients attending the Clinic of Maxillofacial Surgery, University Hospital Brno, in 2020–2022 based on the inclusion and exclusion criteria presented in Figure 1. More details about the methods used are shown in Appendix B – Supplementary Methods.

To promote the comprehensiveness of the analysis, oral samples were examined for microscopic fungi and selected oncogenic viruses (CMV and EBV). Firstly, the bacteriome characteristics of four patient-matched matrices (tumour surface, buccal swabs, tongue swabs, and supragingival dental plaque samples) were compared irrespective of the tumour location. Secondarily, bacteriomes of the swabs from the tumour surface from the corresponding healthy mucosa (healthy tongue swab for tongue tumours, buccal swab for tumours of other locations) were compared using paired analysis.

### Sample collection, laboratory, and data analyses

Multiple oral swab samples were obtained in line with the previously published protocol 21 from the oral cavity of each participating patient during the initial examination using FLOQSwabs^®^ (COPAN, Italy). Swabs were collected from the tumour surface, surface of the healthy buccal mucosa, healthy mucosa from the dorsum of the tongue, and from supragingival plaque. They were immediately deposited in a sterile Eppendorf tube and stored at -80 °C until DNA isolation (DNA Mini Kit, Qiagen, Germany) and subsequent 16S rRNA amplicon sequencing (Miseq Reagent Kit V3, MiSeq instrument, Illumina, USA). The bacteriome and bioinformatic analyses were described in detail previously [22].

An additional swab from the healthy buccal mucosa was placed in a tube with Amies medium (Transystem^TM^, COPAN, Italy) and transported at 4 °C for microbiological examination of microscopic fungi as described previously [23]. The presence of microscopic fungi was monitored using conventional culture techniques; subsequently, the isolated strains were identified using matrix-assisted laser desorption ionization–time of flight mass spectrometry (Bruker, Switzerland).

Next, a biopsy was taken from each tumour, fixed in formalin, and embedded in paraffin for subsequent conventional histopathological examination and determination of p16 protein expression as a previously proposed surrogate marker correlating with the HPV status [8,24], although the sensitivity and specificity were reported to be low in some studies [25–27]. Importantly, however, HPV positivity is generally rare (<5%) in OSCC [25,28,29]. The bioptic samples in our study were also examined for the presence of two other oncogenic viruses, namely, CMV and EBV. For more information on laboratory analyses and statistical analysis, see Appendix B of the Supplementary Methods.

The ratios of the relative abundances of anaerobic to aerobic bacterial genera (anaerobes/aerobes ratio) and gram-negative to gram-positive bacterial genera (G−/G+ ratio) were computed based on the top 25 bacterial genera (i.e. the 25 most abundant genera, which, on average, comprised 91% of the bacteriome composition) across all samples. Furthermore, we introduced a novel bacteriome-based oral dysbiosis index (bbODI), representing the ratio of seven selected gram-negative anaerobic genera belonging to orange/red complex periopathogens [13] (some of which were previously associated with OSCC) [30,31] to seven genera considered to be common bacterial genera associated with healthy oral mucosa [19,32]. Specifically, the bbODI was defined as the sum of the relative abundances of the genera from the orange (*Fusobacterium*, *Prevotella*, *Parvimonas*, and *Peptostreptococcus*) and red (*Porphyromonas*, *Tannerella*, and *Treponema*) complex bacteria, divided by the sum of the relative abundances of selected gram-positive genera (*Streptococcus*, *Veillonella*, *Rothia*, *Gemella*, *Actinomyces*, and *Granulicatella*) and a gram-negative genus (*Haemophilus*). Higher values of this index indicate a relative shift toward potentially pathogenic taxa.

## Results

In total, 84 samples from 21 patients (15 men and 6 women, mean age 62 ± 11.3 years) diagnosed with OSCC were enroled in the study. Only one man and five women were non-smokers. While six patients suffered from OSCC of the tongue, 15 patients suffered from tumours located in other sites of the oral cavity, specifically the maxillary alveolus (four patients), mandibular alveolus (six patients), buccal mucosa (one patient), floor of the mouth (one patient), and trigone retromolar (three patients). Patients with OSCC of the tongue were diagnosed more commonly in early stages than patients with OSCC located at other sites (*p*_*ADJ*_ = 0.025, Fisher test; for basic characteristics, see Supplementary Figure S1). No difference in sex, age, dentition, oral hygiene, smoking, alcohol, metastasis in regional lymph nodes, extranodal extension, grade of tumour differentiation, depth of invasion, perineural invasion, bone invasion, or *Candida* sp. presence was detected (*p*_*ADJ*_ > 0.05 for all variables). With respect to microscopic fungi, only *Candida albicans* was found by culture in five patients. All samples were negative for CMV and EBV as well as for p16 (data not shown).

### Comparison of the oral bacteriomes in four different sites of the oral cavity in patients with OSCC

**Figure 2.**
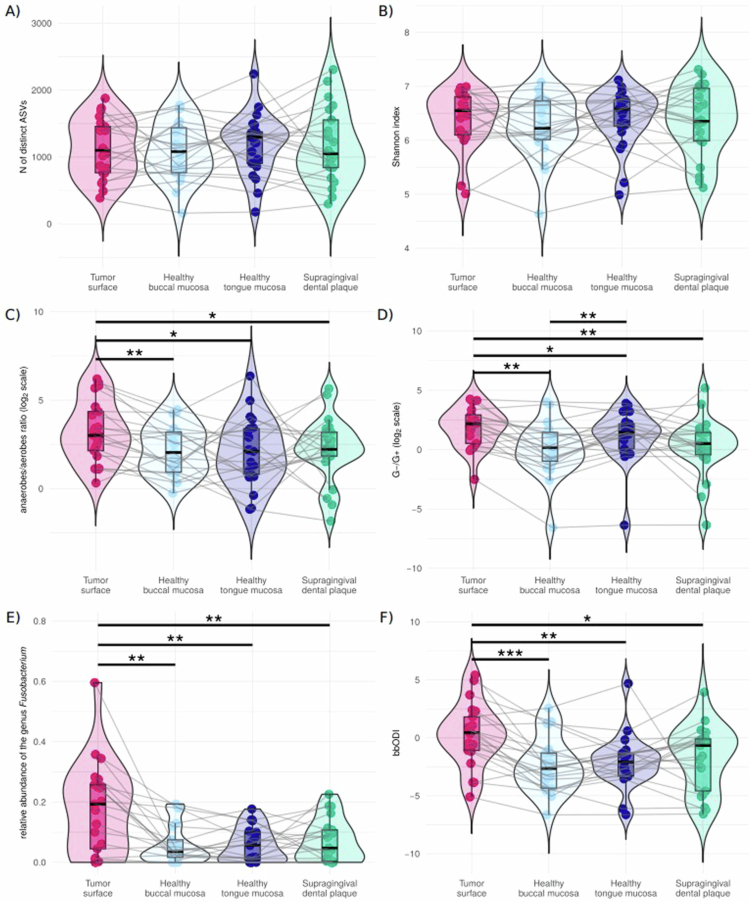
Comparison of selected bacteriome characteristics of four different sites of the oral cavity in patients with oral squamous cell carcinoma (*N* = 21). (A) *N* of distinct ASVs, (B) Shannon index, (C) anaerobes/aerobes ratio (a ratio of relative abundances of anaerobic to aerobic bacterial genera, log_2_ scale), (D) G-/G+ ratio (a ratio of relative abundances of gram-negative to gram-positive bacterial genera, log_2_ scale), (E) relative abundance of the genus *Fusobacterium*, (F) bbODI. Patient-matched samples are connected with grey lines. *N*, number; ASV, amplicon sequence variant; bbODI, bacteriome-based Oral Dysbiosis Index, a ratio of relative abundances of selected gram-negative anaerobic bacteria to selected bacteria common on healthy oral mucosa, see text for details; ٭, *p*_*ADJ*_ < 0.05; ٭٭, *p*_*ADJ*_ < 0.01; ٭٭٭, *p*_*ADJ*_ < 0.001, Wilcoxon paired test.

The sequencing depths were similar between the patient-matched samples from all four sites (*p*_*ADJ*_ > 0.05). The bacteriome *α*-diversity indices (number of distinct ASVs and Shannon index) from individual matrices did not differ (*p*_*ADJ*_ > 0.05 for all, Figure 2A, Figure 2B, and Supplementary Figure S2).

**Figure 3.**
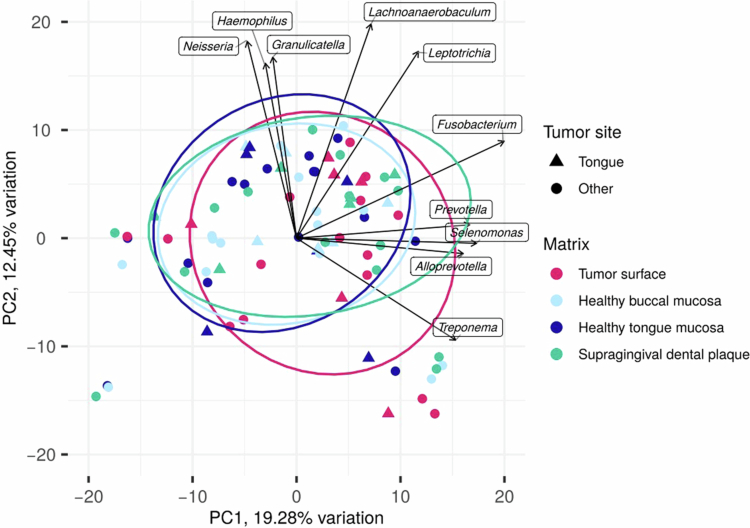
Principal component (PC) analysis for bacteriome composition of four different sites in the oral cavity of patients with oral squamous cell carcinoma (*N* = 21). Tumour site – other, maxillary alveolus/mandibular alveolus/buccal/floor of the mouth/trigone retromolar.

To ensure that it was possible to consider the samples from all tumour sites as one group for further analysis, we performed a principal component analysis of bacteriome compositions (Figure 3). As swabs collected from patients with tumours affecting the tongue were not used, for the most part, separately from swabs originating from patients with tumours in other sites, we were able to perform further analyses based on the full dataset of 21 patients.

A heatmap was created to display complex information about the oral bacteriome characteristics of four different sites in patients with OSCC (Supplementary Figure S3). Hierarchical clustering revealed that, in most cases, buccal and supragingival dental plaque samples were mutually similar, while tumour swabs typically formed distinct clusters, occasionally grouping with supragingival dental plaque samples. Interestingly, in some patients, all (or most) sample types showed high similarity (e.g. patients with ID SP015, SP017, SP020, SP021, and SP028), suggesting that the oral bacteriomes in these individuals exhibited distinct and individualized profiles.

The anaerobes/aerobes ratio statistically significantly differed between the tumour surface and other sample types (*p*_*ADJ*_ < 0.05, Figure 2C). The G-/G+ ratios also differed between the tumour surface and other sample types; moreover, the G-/G+ ratio of healthy tongue swabs was higher than that of healthy buccal mucosa samples (*p*_*ADJ*_ < 0.01, Figure 2D).

**Figure 4.**
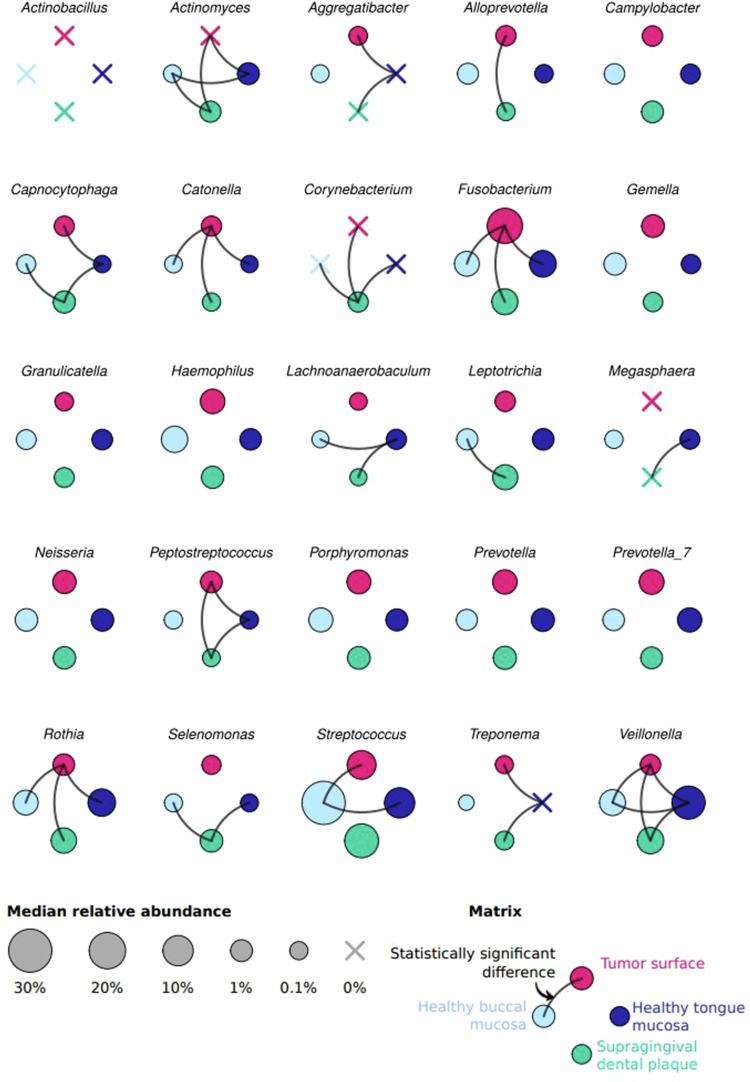
Differences in relative abundances (indicated by dot size) of the 25 most abundant bacterial genera between the swabs from the four investigated matrices (color-coded) from the oral cavity of patients with oral squamous cell carcinoma (*N* = 21). Lines connecting matrices indicate statistically significant differences (*p*_*ADJ*_ < 0.05, Wilcoxon paired test).

Bacterial genera with the highest median relative abundances in the individual matrices are presented in the graphical abstract and in Figure 4. A comparison of the relative abundances of the top 25 bacterial genera among various sites of the oral cavity in patients with OSCC is presented in Supplementary Figure S2. In mutual comparisons of patient-matched non-tumour samples, differences were observed between the supragingival dental plaque vs at least one healthy mucosal sample in the relative abundances of *Actinomyces*, *Aggregatibacter*, *Capnocytophaga*, *Corynebacterium*, *Lachnoanaerobaculum*, *Leptotrichia*, *Megasphaera*, *Peptostreptococcus*, *Selenomonas*, *Treponema,* and *Veillonella*. The relative abundances of the genus *Fusobacterium* and the bbODI were significantly higher on the tumour surface than on the healthy buccal mucosa, healthy tongue, and supragingival dental plaque (*p*_*ADJ*_ < 0.05 for both parameters, Figure 2E, Figure 2F, and Supplementary Figure S2).

### Comparison of oral bacteriome characteristics of the tumour surface and corresponding healthy mucosa swabs

**Figure 5.**
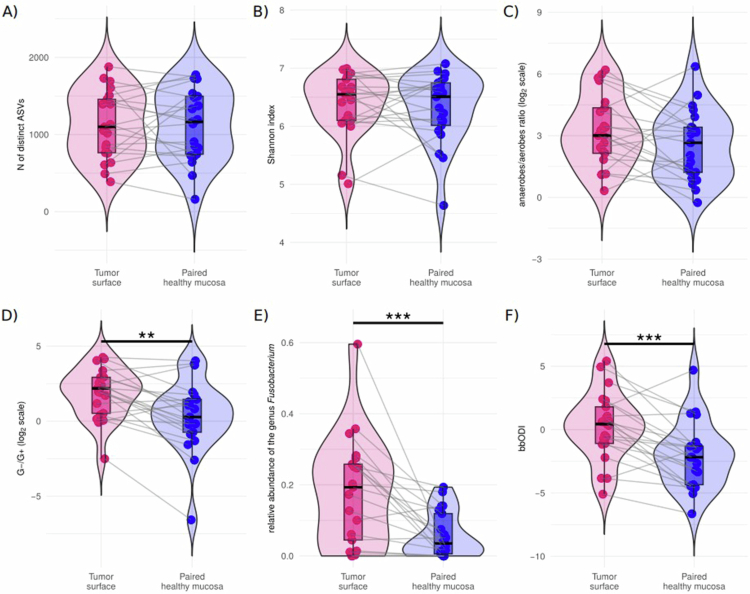
Pairwise comparison of selected oral bacteriome characteristics on the tumour surface and corresponding healthy mucosa surface from patients with oral squamous cell carcinoma (*N* = 21). (A) *N* of distinct ASVs, (B) Shannon index, (C) anaerobes/aerobes ratio (a ratio of relative abundances of anaerobic to aerobic bacterial genera, log_2_ scale), (D) G-/G+ ratio (a ratio of relative abundances of gram-negative to gram-positive bacterial genera, log_2_ scale), (E) relative abundance of the genus *Fusobacterium*, (F) bbODI. Patient-matched samples are connected with grey lines. *N*, number; ASV, amplicon sequence variant; bbODI, bacteriome-based Oral Dysbiosis Index, a ratio of relative abundances of selected gram-negative anaerobic bacteria to selected bacteria common on healthy oral mucosa, see text for details; ٭, *p* < 0.05; ٭٭, *p* < 0.01; ٭٭٭, *p* < 0.001, Wilcoxon paired test.

Sequencing depths were similar between patient-matched samples from the tumour surface and corresponding healthy mucosa (*p*_*ADJ*_ > 0.05). No differences in *α*-diversity (number of distinct ASVs and Shannon index, see Figure 5A, Figure 5B, *p*_*ADJ*_ >  0.05 for both) were detected between swabs from the tumour surface and patient-matched corresponding healthy mucosa. While the paired analysis revealed no difference in the anaerobes/aerobes ratios between the tumour surface and corresponding healthy mucosa swabs (*p*_*ADJ*_ > 0.05, Figure 5C), the G-/G+ ratio was significantly higher on the tumour surface (*p* < 0.01, Figure 5D; *p*_*ADJ*_ > 0.05, Supplementary Figure S4).

**Figure 6.**
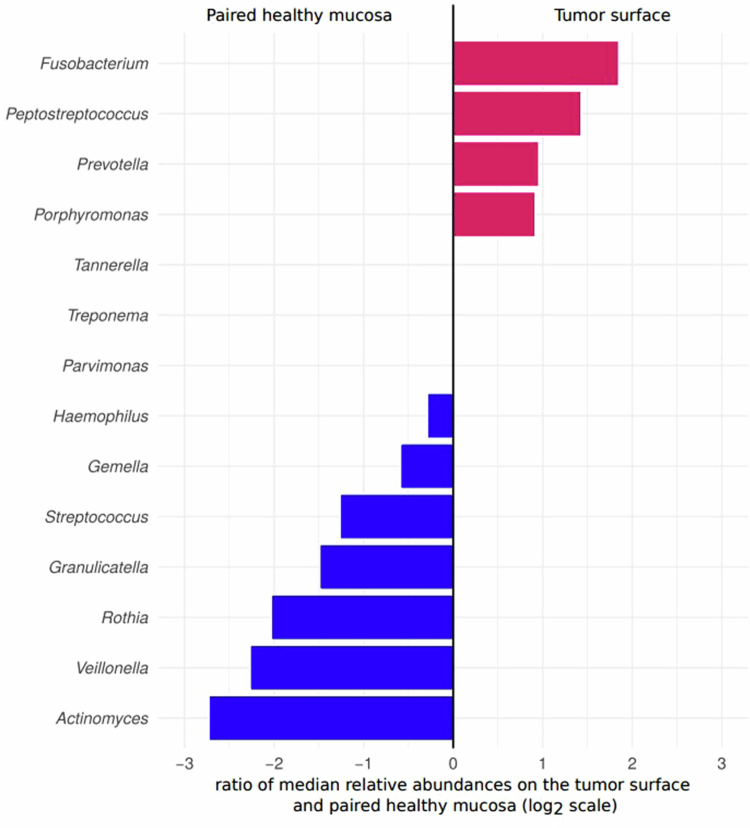
Pairwise comparison of relative abundances of 14 bacterial genera included in the bacteriome-based Oral Dysbiosis Index (bbODI) on tumor and corresponding healthy mucosa surfaces from patients with oral squamous cell carcinoma (*N* = 21).

Among the top 25 bacterial genera, only the relative abundance of genus *Fusobacterium* was significantly higher on the tumour surface compared to the corresponding healthy mucosa samples (*p* < 0.001, Figure 5E; *p*_*ADJ*_ = 0.015, Supplementary Figure S4). A pairwise comparison between the relative abundances of 14 bacterial genera included in bbODI located on the tumour surface and corresponding samples of the healthy mucosa is presented in Figure 6, showing higher relative abundances of four bacterial genera belonging to the orange/red complex periopathogens (i.e. *Fusobacterium*, *Peptostreptococcus*, *Prevotella*, and *Porphyromonas*) on the tumor surface than on the patient-matched healthy mucosa. On the other hand, the relative abundances of all seven bacterial genera used in the denominator of bbODI (i.e. *Actinomyces*, *Veillonella*, *Rothia*, *Granulicatella, Streptococcus, Gemella*, and *Haemophilus*) were higher on patient-matched healthy mucosa than on the tumor surface. This is in accordance with the fact that higher bbODI values were associated with the tumor surface compared to the patient-matched healthy mucosa (*p*_*ADJ*_ < 0.001, Figure 5F; *p*_*ADJ*_ < 0.05 Supplementary Figure S4).

**Figure 7.**
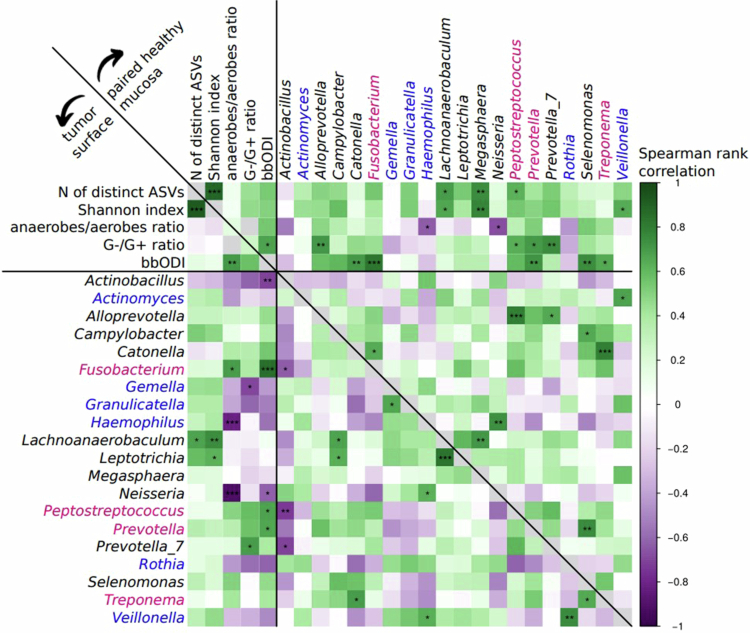
Heatmap of Spearman rank correlations among bacteriome characteristics on the tumor surface (lower triangle) and on the patient-matched healthy oral mucosa (upper triangle) in patients with oral squamous cell carcinoma (*N* = 21). Statistical significance is indicated in individual cells according to the *p* values (٭, *p*_*ADJ*_ ≤ 0.05; ٭٭, *p*_*ADJ*_ ≤ 0.01; ٭٭٭, *p*_*ADJ*_ ≤ 0.001). *N*, number; ASV, amplicon sequence variant; anaerobes/aerobes ratio, a ratio of relative abundances of anaerobic to aerobic bacterial genera; G-/G+ ratio, a ratio of relative abundances of gram-negative to gram-positive bacterial genera; bbODI, bacteriome-based Oral Dysbiosis Index, a ratio of relative abundances of selected gram-negative anaerobic bacteria (magenta) to selected bacteria common on healthy oral mucosa (blue), see text for details.

The bbODI significantly correlated with the relative abundance of the genus *Fusobacterium* on both tumour surfaces (*r* = 0.86, *p*_*ADJ*_ < 0.001) and patient-matched healthy mucosa (*r* = 0.82, *p*_*ADJ*_ < 0.01). Notably, bbODI was significantly positively correlated with the anaerobes/aerobes ratio on the tumour surface (*r* = 0.71, *p*_*ADJ*_ < 0.01), but not with the patient-matched healthy mucosa. Conversely, a significant positive correlation of bbODI with the G-/G+ ratio was observed on the patient-matched healthy mucosa (r = 0.67, *p*_*ADJ*_ < 0.05) but not on the tumour surface. Interestingly, the relative abundances of the gram-negative bacterial genera *Haemophilus* and *Neisseria* both strongly negatively correlated with the anaerobes/aerobes ratio on the tumour surface (*r* = -0.81 and *r* = -0.92, respectively, *p*_*ADJ*_ < 0.001 for both), see Figure 7.

## Discussion

In line with previous studies, we found that the genus *Fusobacterium* is significantly associated with OSCC [12,20,30,31,33,34]. This genus is well-recognized in the literature as a potential driver of carcinogenesis [35,36]. A recent study on OSCC and oral leukoplakias with different degrees of dysplasia reported a higher abundance of *Fusobacterium* sp. in OSCC and oral leukoplakia with high-grade dysplasia compared to those with low-risk grades [37].

Moreover, our study also confirmed the higher abundance of other orange/red complex bacterial genera, such as *Prevotella*, *Porphyromonas* [38,39], or *Peptostreptococcus* [18] in OSCC swabs compared to healthy oral mucosa samples (although these differences were not statistically significant after adjustment). A study in Taiwanese men also reported that tumour sites harboured higher relative abundances of the genera *Fusobacterium*, *Peptostreptococcus*, *Prevotella*, and others than their contralateral normal controls, whereas a decrease in the relative abundance of *Streptococcus* spp. was detected on the surface of OSCC lesions [40]. These results are consistent with the conclusions of a systematic analysis reporting a higher abundance of *Fusobacterium, Peptostreptococcus, Prevotella,* and others in HNSCC and, on the contrary, depletion of *Streptococcus, Actinomyces, Veillonella,* and *Rothia* in tumour samples [41]. The authors of that paper proposed that oral microbial dysbiosis could serve as a promising tool for HNSCC screening and diagnosis.

In our study, we attempted to address this issue by introducing the bbODI, combining the relative abundance of multiple potentially pathogenic bacterial genera and common bacterial genera of healthy oral mucosa into a single parameter. While significant differences between the tumour surface and patient-matched healthy mucosa were observed not only in bbODI but also in the relative abundance of *Fusobacterium*, bbODI has several methodological and biological advantages over the use of just this single genus. Firstly, the bbODI describes the overall change in the cumulative relative abundances of potentially pathogenic and commensal taxa more comprehensively, mitigating the impact of variability or measurement error in any single taxon and enhancing the robustness of the analysis (thus reducing the susceptibility to outliers). Secondly, it produces an approximately normal distribution, enabling the application of parametric statistical methods with greater power and interpretability. It should be noted that several genera included in the bbODI, such as *Parvimonas* and *Tanerella*, have not been detected in our study (or have been detected in negligible amounts). From this perspective, it may appear that they could be disregarded in our group of patients. However, as they have been added because of their biological relevance, on which the bbODI was based (they are part of the orange/red complexes), we still assume that their inclusion in the index is justified and might possibly prove beneficial in other populations.

The bbODI positively correlated not only with the relative abundances of the genus *Fusobacterium*, but also with both investigated ratios (anaerobes/aerobes and G-/G+), confirming that this index provides comprehensive information on bacterial composition. The analysis of correlations between the ratios and relative abundances of individual genera revealed that, unsurprisingly, the genera *Haemophilus* (a gram-negative facultative anaerobe) and *Neisseria* (a gram-negative aerobe) were negatively correlated with the anaerobes/aerobes ratio, particularly on the tumour surface, which suggests that the decline in these two bacterial genera can be a driver of the change in the anaerobes/aerobes ratio. Moreover, *Haemophilus*, which is generally considered a commensal bacterial genus in the oral cavity [32], was relatively more abundant on swabs of corresponding healthy mucosa than on the tumour surface in the paired analysis. Based on the above, we hypothesize that the genus *Haemophilus* is one of the key elements of the eubiotic state in the oral environment and that replacing it with orange/red complex bacteria, which have similar needs in terms of living conditions, leads to a dysbiotic state associated with OSCC.

Several oral dysbiosis indices have been proposed previously in association with various diagnoses (periodontitis, recurrent aphthous stomatitis, OSCC, or oropharyngeal squamous cell carcinoma – OPSCC; see Appendix C – Supplementary Table 1) [6–10]. Only three of those, however, attempted to establish ODIs based on 16S rRNA amplicon sequencing data to predict the risk of development or the prognosis of OSCC (or OPSCC). These case‒control studies or case series used ODIs (named Microbial Dysbiosis Index, MDI [9,10], or Oral Microbiome Biomarker Panel [8] to predict OSCC and OPSCC) based on bacteriome compositions of formalin-fixed paraffin blocks (FFPE) of tumour tissue [9], saliva [10], or oral rinse samples [8]. These studies, therefore, were not designed to compare the local bacteriomes of the tumorous and healthy mucosa. Interestingly, the relative abundances of the genus *Fusobacterium* did not play a role in the OSCC ODIs from these studies, while this genus dominated in our bb-ODI. This may be associated with the paired design used in our study – without the within-individual comparison of the potentially pathogenic and healthy site, a locally higher abundance of the genus *Fusobacterium* may remain unrevealed. Besides, the fact that the three aforementioned studies investigated different populations (Chinese, Australian, or mixed population from the USA), while our study focused on a genetically homogeneous Central European population, which may have also influenced the differences in the bacteriome profiles in their studies and ours.

To this date, no study has conducted a paired (within-patient) comparison between the bacteriomes of supragingival dental plaque and the tumour surface. Of available studies assessing the bacterial composition of the supragingival dental plaque in patients with OSCC, two compared only bacteriomes of this matrix between patients with OSCC and healthy controls 39,42. There are also three studies analyzing supragingival dental plaque vs. saliva in patients with OSCC. Of those, two analyzed very small study groups (five patients [43] and ten patients [44], respectively). The last one found that in the supragingival dental plaque, the relative abundance of *F. nucleatum* (and some other species) was higher than that in the saliva (but their results were not adjusted for multiple comparisons) [20].

Our study is the first to analyze the differences between the tumour surface, healthy mucosa swabs, and supragingival dental plaque. Expectably, there were differences between the relative abundance of certain individual bacterial genera between the supragingival dental plaque and healthy mucosal samples. Of the relative abundances of the major bacteria of the orange or red complexes, differences between supragingival dental plaque and healthy mucosal samples were observed only for *Peptostreptococcus* and *Treponema*. Notably, the relative abundances of the G-/G+ ratios, the genus *Fusobacterium,* and bbODI were all increased on the tumour surface compared to the patient-matched supragingival dental plaque samples. Nevertheless, grouping according to bacteriome compositions revealed that tumour swabs were more commonly grouped with supragingival dental plaque samples than with healthy oral mucosa samples.

Previously, Chang et al. [45] demonstrated a positive correlation of *Porphyromonas gingivalis* and *F. nucleatum* between the OSCC tissue and subgingival plaque specimens, suggesting that subgingival pockets can act as a reservoir of the tumorigenic bacteria. However, the direction of causality is not entirely clear. Even though these orange/red complex bacteria are well-known to be associated with OSCC [36] and its progression [46], the explanation might lie not just in their tumorigenic potential, but also in the formation of a favourable environment for these bacteria on the tumour surface. The normal oral mucosa, including the tongue, lips, and floor of the mouth, is characterized by crypts–small invaginations or pits. In OSCC, these crypts can exhibit abnormal growth, thus forming deeper crevices and a suitable environment for anaerobic bacteria, similar to the subgingival pockets. In effect, further investigations are needed to determine whether these bacteria are a causative agent in OSCC, or if they rather simply tend to colonize the tumour tissue [47].

In addition to the supragingival dental plaque and healthy buccal mucosa, we also assessed the tongue to obtain a complex view of the bacteriome across the entire oral cavity. This is important as different sites of the oral cavity have been shown to differ in bacterial composition [48]. In our study, a higher relative abundance of gram-negative bacteria was detected in swabs from healthy tongue mucosa than in buccal swabs. This was expected because of the differences in the morphology of the healthy tongue surface and buccal mucosa. In addition, we observed that 25% of patients with OSCC had oral candidosis, and none of them was positive for CMV or EBV.

Although the number of patients included in the study may have been relatively small, the study group size was designed in line with the ‘rule of thumb’ [49] for microbiome studies and proved to be sufficient for detecting statistically significant differences between individual matrices in multiple parameters. The inclusion of patients with OSCC tumours in various sites might also appear to be a limitation of the study. However, the principal component analysis revealed no grouping of samples relative to the tumour sites, which allowed us to draw more generally valid conclusions about the bacteriomes of the tumour surface and the healthy oral mucosa in patients with OSCC, as well as better judge the applicability of the proposed bbODI parameter. On the other hand, sampling from multiple sites of the oral cavity, including the tongue (which is unique among studies conducted so far) and supragingival dental plaque, is a major strength of our study. Pending validation by further research, the bbODI might be implemented in secondary and tertiary prevention, serving as a predictive tool for the early detection of OSCC development or recurrence. Recent research trends indicate a growing interest in the therapeutic potential of the oral microbiome within the scope of OSCC management. In this respect and upon broader validation, bbODI could reveal the presence of a higher-risk oral microbiome, and, together with the knowledge of the bacteriome profile, could guide optimized treatment strategies.

Other novel methods—such as exhaled breath analysis based on the detection of volatile compounds produced by the tumour environment–have also shown promise in diagnosing some cancers, especially those of the aerodigestive tract [50,51]. A side-by-side comparison of the screening performance of these non-invasive techniques, i.e. bbODI and exhaled breath analysis, may represent a promising area for future research. Future studies on the topic of oral dysbiosis and bbODI as biomarkers of the risk for OSCC development could also consider enroling a matched group of patients without OSCC as controls, including samples from different sites of the oral cavity, i.e. subgingival plaque samples. To pave the way for the potential diagnostic use of bacteriome differences between healthy mucosa and OSCC, as suggested by Ting et al. [41], it would also be necessary to establish differences between the microbial compositions of healthy mucosa and precancerous lesions (and between these lesions and OSCC surface).

## Conclusions

Our study revealed that the tumour surface in patients with OSCC is associated with a higher relative abundance of the genus *Fusobacterium,* G-/G+ ratio, and a newly introduced bacteriome-based oral dysbiosis index (bbODI) compared to samples from other oral sites (healthy buccal and tongue mucosa, supragingival dental plaque). The bbODI provides a comprehensive single-value characteristic of the bacteriome and captures broader ecological shifts in the oral microbiome, reflecting a community-level imbalance between pathogenic and commensal bacteria rather than the change in a single genus. In this way, it provides a more robust and complex measure of bacteriome dysbiosis in the oral cavity.

## Supplementary Material

Appendix A_Supplementary Figures.pdf Appendix A_Supplementary Figures.pdf

Appendix_B_Supplementary_Methods_.docx Appendix_B_Supplementary_Methods_.docx

Appendix C_Supplementary Table 1.docx Appendix C_Supplementary Table 1.docx

## Data Availability

The data for this study have been deposited in the European Nucleotide Archive (ENA) at EMBL-EBI under accession number PRJEB98225 (https://www.ebi.ac.uk/ena/browser/view/PRJEB98225).

## A preprint of this manuscript is available:

David Szaraz, Jan Bohm, Ctirad Machacek et al. Bacteriome-Based Oral Dysbiosis Index in Patients with Oral Squamous Cell Carcinoma, available at Research Square [https://doi.org/10.21203/rs.3.rs-7724435/v1].
