# Supplementary material for: Bacteriome-based oral dysbiosis index in patients with oral squamous cell carcinoma
Source: J Oral Microbiol. 2026 May 7;18(1):2668149. doi: 10.1080/20002297.2026.2668149 (PMC13159579; doi:10.1080/20002297.2026.2668149)
Supplement: Appendix A_Supplementary Figures.pdf [file ZJOM_A_2668149_SM6716.pdf]

	Tumor site		
	Tongue<br>N = 6 (28.6%)	Other<br>N = 15 (71.4%)	*p*-value
**Sex**			
Male	4 (66.67%)	11 (73.33%)	1
Female	2 (33.33%)	4 (26.67%)	
**Age [years]**			
five number summary	[45 | 49 | <u>57</u> | 66 | 66]	[46 | 54.5 | <u>60</u> | 72 | 86]	1
mean ± sd	56.7 ± 8.6	64.2 ± 11.8	1
**Oral hygiene**			
Poor	3 (50%)	4 (26.67%)	1
Good	3 (50%)	11 (73.33%)	
**Smoking**			
Smoker	5 (83.33%)	9 (60%)	1
Ex-smoker	0 (0%)	1 (6.67%)	
Non-smoker	1 (16.67%)	5 (33.33%)	
**Alcohol**			
Teetotal	1 (16.67%)	4 (26.67%)	1
Ex-drinker	1 (16.67%)	0 (0%)	
Occasional drinker	3 (50%)	4 (26.67%)	
Drinker	1 (16.67%)	7 (46.67%)	
**Clinical staging**			
I or II	5 (83.33%)	1 (6.67%)	**0.025**
IVA or IVB	1 (16.67%)	14 (93.33%)	
**Metastasis in regional lymph nodes**			
No	5 (83.33%)	5 (33.33%)	0.698
Yes	1 (16.67%)	10 (66.67%)	
**Extranodal extension**			
No	2 (33.33%)	12 (80%)	0.367
Yes	0 (0%)	1 (6.67%)	
Unknown	4 (66.67%)	2 (13.33%)	
**Grade of tumor differentiation**			
Well	4 (66.67%)	8 (53.33%)	1
Moderately	0 (0%)	6 (40%)	
Poorly	2 (33.33%)	1 (6.67%)	
**Tumor size**			
<4 cm	5 (83.33%)	3 (20%)	0.195
≥4 cm	1 (16.67%)	12 (80%)	
**Depth of invasion**			
<4 mm	1 (16.67%)	3 (20%)	1
4-10 mm	3 (50%)	5 (33.33%)	
≥10 mm	1 (16.67%)	7 (46.67%)	
Unknown	1 (16.67%)	0 (0%)	
**Perineual invasion**			
No	4 (66.67%)	11 (73.33%)	1
Yes	0 (0%)	3 (20%)	
Unknown	2 (33.33%)	1 (6.67%)	
**Bone invasion**			
No	2 (33.33%)	2 (13.33%)	0.247
Yes	0 (0%)	10 (66.67%)	
Unknown	4 (66.67%)	3 (20%)	
***Candida* sp. presence**			
No	4 (66.67%)	11 (73.33%)	1
Yes	1 (16.67%)	4 (26.67%)	
Missing	1 (16.67%)	0 (0%)	

Five-number summary stands for [minimum | 1st quartile | <u>median</u> | 3rd quartile | maximum].
To test difference in medians a Wilcoxon test or non-parametric ANOVA (Kruskal-Wallis test) was used.
To test independence of a categorical variables a Fisher test with Monte-Carlo simulation (10^5 replicates) was used.
All *p*-values were adjusted using Benjamini & Hochberg method.

Supplementary Figure S1. Demographic, clinical, and microbiological characteristics of patients with oral squamous cell carcinoma (N = 21).

N, number; Missing, data is missing due to a problem with sample transport; Unknown, not examined; Tumor site – Other, maxillary alveolus/mandibular alveolus/buccal/floor of the mouth/ trigone retromolar.

	Matrix				
	Tumor surface N = 21 (25%)	Healthy buccal mucosa N = 21 (25%)	Healthy tongue mucosa N = 21 (25%)	Supragingival dental plaque N = 21 (25%)	*p*-value
**N of distinct ASVs**					
five-number summary	[387 | 764 | 1100 | 1457 | 1878]	[160 | 767 | 1082 | 1437 | 1775]	[178 | 905 | 1296 | 1375 | 2242]	[298 | 842 | 1046 | 1555 | 2308]	1
**Shannon index**					
five-number summary	[5 | 6.1 | 6.5 | 6.8 | 7]	[4.6 | 6 | 6.2 | 6.7 | 7.1]	[5 | 6.3 | 6.6 | 6.8 | 7.1]	[5.1 | 6 | 6.4 | 7 | 7.3]	1
**anaerobes/aerobes ratio**					
five-number summary	[0.3 | 2.1 | 3 | 4.4 | 6.2]	[-0.3 | 0.9 | 2 | 3.2 | 4.5]	[-1.2 | 0.8 | 2.1 | 3.4 | 6.4]	[-1.8 | 1.8 | 2.2 | 3.2 | 5.7]	0.084
**G-/G+ ratio**					
five-number summary	[-2.5 | 0.5 | 2.2 | 2.9 | 4.2]	[-6.6 | -1 | 0.2 | 1.5 | 4]	[-6.4 | 0.1 | 1.5 | 2.2 | 3.9]	[-6.4 | -0.4 | 0.5 | 1.4 | 5.2]	**0.006**
**bbODI**[1]					
five-number summary	[-5.1 | -1.1 | 0.4 | 1.8 | 5.4]	[-6.6 | -4.3 | -2.6 | -1.3 | 2.6]	[-6.6 | -3.3 | -2.1 | -1.4 | 4.7]	[-6.6 | -4.6 | -0.7 | -0.1 | 3.9]	**0.003**
***Actinobacillus* [%]**					
five-number summary	[0 | 0 | 0 | 0 | 22.8]	[0 | 0 | 0 | 0 | 10.1]	[0 | 0 | 0 | 0 | 2.5]	[0 | 0 | 0 | 0 | 3.2]	1
***Actinomyces* [%]**					
five-number summary	[0 | 0 | 0 | 0.3 | 0.8]	[0 | 0 | 0.1 | 0.5 | 2.6]	[0 | 0.3 | 1.3 | 2 | 5.5]	[0 | 0.4 | 1 | 1.9 | 10.9]	**0.001**
***Aggregatibacter* [%]**					
five-number summary	[0 | 0 | 0.2 | 1 | 20]	[0 | 0 | 0.1 | 0.4 | 3.5]	[0 | 0 | 0 | 0.2 | 1.4]	[0 | 0 | 0 | 0.7 | 6.6]	0.096
***Alloprevotella* [%]**					
five-number summary	[0 | 0.2 | 0.7 | 2.7 | 11.7]	[0 | 0 | 0.9 | 1.6 | 14.2]	[0 | 0 | 0.2 | 3.6 | 15.6]	[0 | 0 | 0.2 | 1.1 | 3.1]	0.237
***Campylobacter* [%]**					
five-number summary	[0 | 0.4 | 0.7 | 2.5 | 6]	[0 | 0.2 | 0.7 | 1.7 | 5]	[0 | 0.1 | 0.5 | 1.5 | 3.6]	[0 | 0.1 | 1.3 | 1.9 | 4.5]	1
***Capnocytophaga* [%]**					
five-number summary	[0 | 0 | 0.6 | 3.1 | 31.9]	[0 | 0.1 | 0.3 | 0.8 | 5.4]	[0 | 0 | 0.1 | 0.5 | 1.1]	[0 | 0.3 | 1.5 | 3 | 10.5]	**0.032**
***Catonella* [%]**					
five-number summary	[0 | 0.1 | 0.6 | 1.6 | 5]	[0 | 0 | 0.1 | 0.4 | 0.8]	[0 | 0 | 0.1 | 0.3 | 2.9]	[0 | 0 | 0.2 | 0.4 | 0.9]	0.066
***Corynebacterium* [%]**					
five-number summary	[0 | 0 | 0 | 0 | 2.9]	[0 | 0 | 0 | 0 | 0.3]	[0 | 0 | 0 | 0 | 0.4]	[0 | 0 | 0.6 | 2.8 | 9.8]	**<0.001**
***Fusobacterium* [%]**					
five-number summary	[0 | 4.5 | 19.3 | 25.8 | 59.6]	[0 | 1.7 | 3.5 | 7.5 | 19.3]	[0 | 0.8 | 5.6 | 10 | 17.7]	[0 | 0.4 | 4.8 | 10.8 | 22.6]	**0.003**
***Gemella* [%]**					
five-number summary	[0 | 0.4 | 1.9 | 2.5 | 4.6]	[0 | 0.5 | 1.5 | 3.7 | 12.8]	[0 | 0.1 | 0.4 | 0.8 | 4.3]	[0 | 0.2 | 0.4 | 2 | 7.4]	0.096
***Granulicatella* [%]**					
five-number summary	[0 | 0 | 0.2 | 0.7 | 4.3]	[0 | 0.1 | 0.5 | 1.9 | 6.8]	[0 | 0.1 | 0.8 | 1.6 | 4.7]	[0 | 0.2 | 0.5 | 0.9 | 3.3]	0.237
***Haemophilus* [%]**					
five-number summary	[0 | 1.6 | 3.3 | 5.5 | 27.4]	[0 | 1.2 | 5.1 | 9.8 | 38.6]	[0 | 0.5 | 1.1 | 5.9 | 48.8]	[0 | 0.2 | 1.5 | 8.4 | 34.5]	0.677
***Lachnoanaerobaculum* [%]**					
five-number summary	[0 | 0 | 0.1 | 0.6 | 3.2]	[0 | 0 | 0.1 | 0.3 | 1.3]	[0 | 0 | 0.6 | 0.8 | 4.2]	[0 | 0 | 0.1 | 0.4 | 3.6]	0.237
***Leptotrichia* [%]**					
five-number summary	[0 | 0.1 | 0.7 | 3 | 18.7]	[0 | 0.2 | 1 | 2.2 | 4.1]	[0 | 0.1 | 1.2 | 2.9 | 10.2]	[0 | 0.3 | 3.8 | 7.9 | 22.8]	0.066
***Megasphaera* [%]**					
five-number summary	[0 | 0 | 0 | 0.2 | 3]	[0 | 0 | 0.2 | 0.7 | 1.4]	[0 | 0 | 0.5 | 1.9 | 15.5]	[0 | 0 | 0 | 0.1 | 1]	0.066
***Neisseria* [%]**					
five-number summary	[0 | 0.4 | 2.2 | 7.2 | 22.9]	[0 | 0.9 | 1.6 | 9.9 | 24.2]	[0 | 0 | 1.7 | 12.5 | 47.7]	[0 | 0.4 | 1.6 | 2.5 | 19.8]	1
***Peptostreptococcus* [%]**					
five-number summary	[0 | 0.1 | 1.2 | 1.6 | 3.2]	[0 | 0 | 0.2 | 0.5 | 9.9]	[0 | 0 | 0.2 | 0.7 | 2.7]	[0 | 0 | 0.1 | 0.2 | 0.8]	**0.008**
***Porphyromonas* [%]**					
five-number summary	[0 | 0.9 | 2.7 | 5.6 | 14.9]	[0 | 0.8 | 2.8 | 5 | 20.8]	[0 | 0.1 | 1.4 | 4.2 | 6.1]	[0 | 0.4 | 1.8 | 6.1 | 65.6]	1
***Prevotella* [%]**					
five-number summary	[0 | 0.8 | 3.1 | 6.7 | 38.2]	[0 | 0.2 | 0.9 | 5.3 | 29.9]	[0 | 0.4 | 2.1 | 4.1 | 48.9]	[0 | 0.5 | 2.1 | 6.9 | 19.1]	1
***Prevotella_7* [%]**					
five-number summary	[0 | 1.2 | 3.5 | 6.4 | 71.9]	[0 | 0.9 | 1.1 | 6.2 | 30.7]	[0 | 1.2 | 3 | 10.7 | 53.4]	[0 | 0.4 | 1.3 | 4.5 | 16.2]	0.209
***Rothia* [%]**					
five-number summary	[0 | 0.2 | 1.2 | 1.7 | 6.2]	[0 | 1.5 | 3.4 | 7.8 | 39.5]	[0 | 2.2 | 6.6 | 14.6 | 31.2]	[0 | 0.6 | 4 | 12.5 | 59.7]	**0.004**
***Selenomonas* [%]**					
five-number summary	[0 | 0 | 0.3 | 3.3 | 9.1]	[0 | 0 | 0.2 | 1.2 | 3.5]	[0 | 0 | 0.1 | 0.4 | 5.2]	[0 | 0.1 | 1.7 | 4.9 | 9.4]	0.066
***Streptococcus* [%]**					
five-number summary	[0.4 | 3.5 | 8.4 | 20.6 | 78.7]	[0.9 | 10.6 | 33.6 | 50.6 | 82.3]	[0.7 | 7.7 | 10.7 | 18.7 | 50.5]	[1.4 | 10.2 | 16.9 | 25 | 66.9]	**0.045**
***Treponema* [%]**					
five-number summary	[0 | 0 | 0.2 | 0.8 | 13.7]	[0 | 0 | 0 | 0.3 | 3.5]	[0 | 0 | 0 | 0 | 3.3]	[0 | 0 | 0.3 | 0.6 | 4.4]	**0.016**
***Veillonella* [%]**					
five-number summary	[0 | 0.3 | 0.7 | 2.8 | 19.1]	[0 | 2.1 | 4.7 | 9.7 | 25.3]	[0 | 10.7 | 15.6 | 22.6 | 65.4]	[0 | 1 | 5.3 | 10.8 | 38.5]	**0.004**

Five-number summary stands for [minimum | 1st quartile | median | 3rd quartile | maximum].
To test difference in medians a Friedman test was used.
All *p*-values were adjusted using Benjamini & Hochberg method.

[1]The **bacteriome-based Oral Dysbiosis Index** represents the $\log_2$ fold change in the total relative abundance of the genera *Fusobacterium*, *Parvimonas*, *Peptostreptococcus*, *Porphyromonas*, *Prevotella*, *Tannarella*, and *Treponema* compared to the genera *Actinomyces*, *Gemella*, *Granulicatella*, *Haemophilus*, *Rothia*, *Streptococcus*, and *Veillonella*. Higher values of the bbODI indicate a greater degree of microbial imbalance (dysbiosis), with an increased relative abundance of potentially pathogenic genera.

N, number; ASV, amplicon sequence variant; anaerobes/aerobes ratio, ratio of relative abundances of anaerobic to aerobic bacterial genera ($\log_2$ scale); G-/G+ ratio, ratio of relative abundances of gramnegative to grampositive bacterial genera ($\log_2$ scale)

Supplementary Figure S2. Comparison of selected bacteriome characteristics in different sites within the oral cavity of patients with oral squamous cell carcinoma (N = 21).

N, number; ASV, amplicon sequence variant.

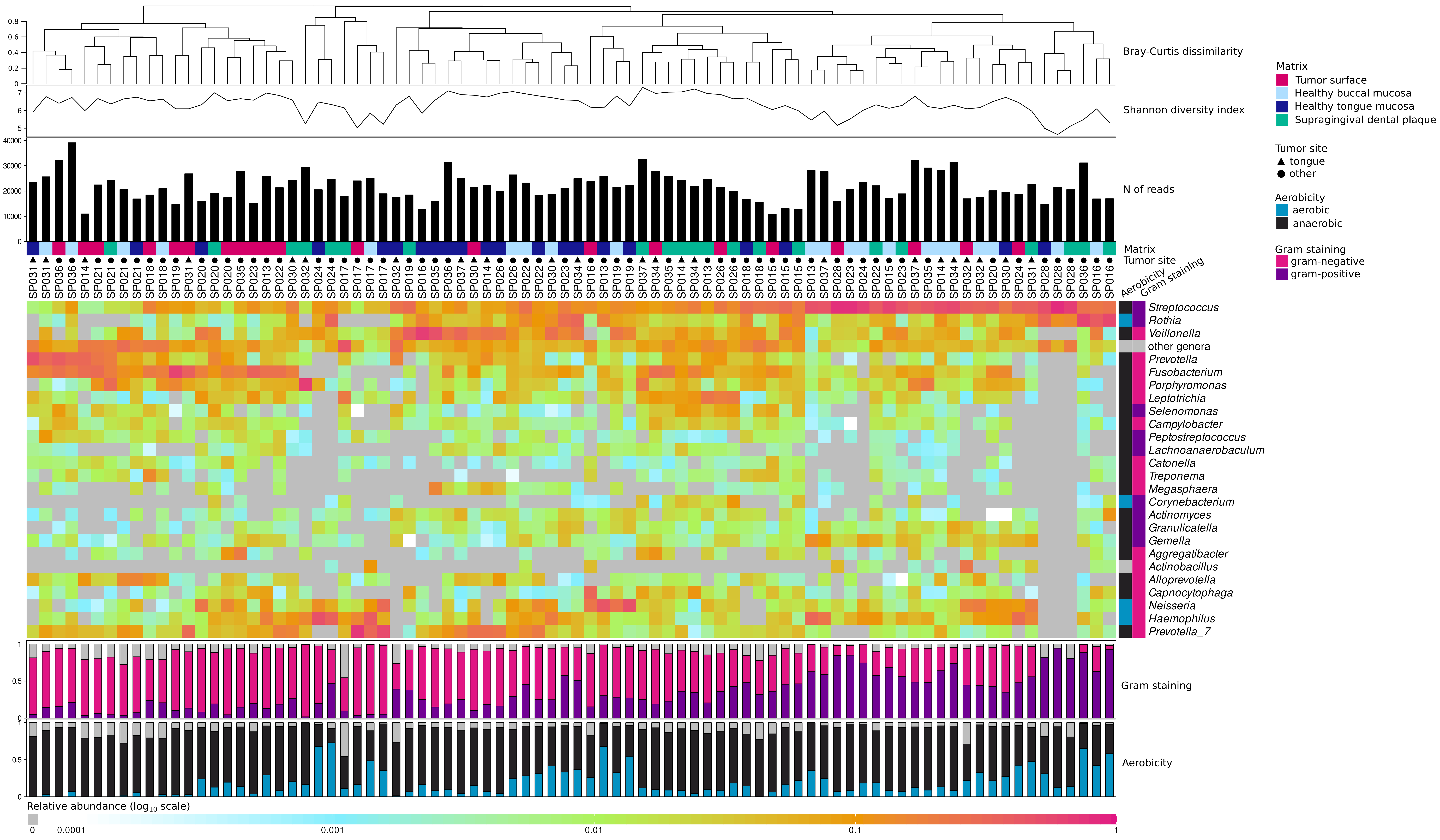

Supplementary Figure S3. Heatmap with bacteriome characteristics.

N, number; SP, individual patient code; Tumor site – Other, maxillary alveolus/mandibular alveolus/buccal/floor of the mouth/ trigone retromolar.

	Matrix		
	Tumor surface<br>N = 21 (50%)	Paired healthy mucosa<br>N = 21 (50%)	*p*-value
**N of distinct ASVs**			
five-number summary	[387 | 764 | 1100 | 1457 | 1878]	[160 | 745 | 1165 | 1497 | 1775]	1
**Shannon index**			
five-number summary	[5 | 6.1 | 6.5 | 6.8 | 7]	[4.6 | 6 | 6.5 | 6.7 | 7.1]	1
**anaerobes/aerobes ratio**			
five-number summary	[0.3 | 2.1 | 3 | 4.4 | 6.2]	[-0.3 | 1.2 | 2.7 | 3.4 | 6.4]	0.426
**G-/G+ ratio**			
five-number summary	[-2.5 | 0.5 | 2.2 | 2.9 | 4.2]	[-6.6 | -0.7 | 0.3 | 1.5 | 4]	0.426
**bbODI**[1]			
five-number summary	[-5.1 | -1.1 | 0.4 | 1.8 | 5.4]	[-6.6 | -4.3 | -2.2 | -1.3 | 4.7]	**0.031**
***Actinobacillus* [%]**			
five-number summary	[0 | 0 | 0 | 0 | 22.8]	[0 | 0 | 0 | 0 | 10.1]	1
***Actinomyces* [%]**			
five-number summary	[0 | 0 | 0 | 0.3 | 0.8]	[0 | 0 | 0.2 | 1.7 | 5.5]	1
***Aggregatibacter* [%]**			
five-number summary	[0 | 0 | 0.2 | 1 | 20]	[0 | 0 | 0 | 0.2 | 1.1]	1
***Alloprevotella* [%]**			
five-number summary	[0 | 0.2 | 0.7 | 2.7 | 11.7]	[0 | 0 | 0.8 | 1.6 | 14.2]	1
***Campylobacter* [%]**			
five-number summary	[0 | 0.4 | 0.7 | 2.5 | 6]	[0 | 0 | 0.9 | 1.7 | 5]	1
***Capnocytophaga* [%]**			
five-number summary	[0 | 0 | 0.6 | 3.1 | 31.9]	[0 | 0 | 0.2 | 0.5 | 5.4]	0.865
***Catonella* [%]**			
five-number summary	[0 | 0.1 | 0.6 | 1.6 | 5]	[0 | 0 | 0.1 | 0.4 | 0.7]	0.335
***Corynebacterium* [%]**			
five-number summary	[0 | 0 | 0 | 0 | 2.9]	[0 | 0 | 0 | 0 | 0.3]	1
***Fusobacterium* [%]**			
five-number summary	[0 | 4.5 | 19.3 | 25.8 | 59.6]	[0 | 0.6 | 3.5 | 11.9 | 19.3]	**0.017**
***Gemella* [%]**			
five-number summary	[0 | 0.4 | 1.9 | 2.5 | 4.6]	[0 | 0.6 | 1.2 | 2.6 | 12.8]	1
***Granulicatella* [%]**			
five-number summary	[0 | 0 | 0.2 | 0.7 | 4.3]	[0 | 0.1 | 0.5 | 1.6 | 3.7]	0.818
***Haemophilus* [%]**			
five-number summary	[0 | 1.6 | 3.3 | 5.5 | 27.4]	[0 | 0.9 | 3.6 | 8.7 | 38.6]	1
***Lachnoanaerobaculum* [%]**			
five-number summary	[0 | 0 | 0.1 | 0.6 | 3.2]	[0 | 0 | 0.1 | 0.6 | 1.8]	1
***Leptotrichia* [%]**			
five-number summary	[0 | 0.1 | 0.7 | 3 | 18.7]	[0 | 0.1 | 0.6 | 2.2 | 4.7]	1
***Megasphaera* [%]**			
five-number summary	[0 | 0 | 0 | 0.2 | 3]	[0 | 0 | 0.2 | 0.7 | 6.2]	0.94
***Neisseria* [%]**			
five-number summary	[0 | 0.4 | 2.2 | 7.2 | 22.9]	[0 | 0.1 | 0.9 | 6.2 | 24.2]	1
***Peptostreptococcus* [%]**			
five-number summary	[0 | 0.1 | 1.2 | 1.6 | 3.2]	[0 | 0 | 0.2 | 0.5 | 3.3]	0.639
***Porphyromonas* [%]**			
five-number summary	[0 | 0.9 | 2.7 | 5.6 | 14.9]	[0 | 0.2 | 1.1 | 5.1 | 20.8]	1
***Prevotella* [%]**			
five-number summary	[0 | 0.8 | 3.1 | 6.7 | 38.2]	[0 | 0.2 | 1.9 | 6.1 | 48.9]	0.818
***Prevotella_7* [%]**			
five-number summary	[0 | 1.2 | 3.5 | 6.4 | 71.9]	[0 | 0.9 | 1.8 | 8.5 | 30.7]	1
***Rothia* [%]**			
five-number summary	[0 | 0.2 | 1.2 | 1.7 | 6.2]	[0 | 1.5 | 3.3 | 7.3 | 39.5]	0.08
***Selenomonas* [%]**			
five-number summary	[0 | 0 | 0.3 | 3.3 | 9.1]	[0 | 0 | 0.1 | 1.3 | 5.2]	1
***Streptococcus* [%]**			
five-number summary	[0.4 | 3.5 | 8.4 | 20.6 | 78.7]	[0.7 | 10.6 | 21.5 | 42.8 | 82.3]	0.426
***Treponema* [%]**			
five-number summary	[0 | 0 | 0.2 | 0.8 | 13.7]	[0 | 0 | 0 | 0.3 | 3.5]	0.94
***Veillonella* [%]**			
five-number summary	[0 | 0.3 | 0.7 | 2.8 | 19.1]	[0 | 2.4 | 5 | 15.9 | 31.3]	0.426

Five-number summary stands for [minimum | 1st quartile | median | 3rd quartile | maximum].
To test difference in medians a Friedman test was used.
All *p*-values were adjusted using Benjamini & Hochberg method.

[1]The **bacteriome-based Oral Dysbiosis Index** represents the $\log_2$ fold change in the total relative abundance of the genera *Fusobacterium*, *Parvimonas*, *Peptostreptococcus*, *Porphyromonas*, *Prevotella*, *Tannarella*, and *Treponema* compared to the genera *Actinomyces*, *Gemella*, *Granulicatella*, *Haemophilus*, *Rothia*, *Streptococcus*, and *Veillonella*. Higher values of the bbODI indicate a greater degree of microbial imbalance (dysbiosis), with an increased relative abundance of potentially pathogenic genera.

N, number; ASV, amplicon sequence variant; anaerobes/aerobes ratio, ratio of relative abundances of anaerobic to aerobic bacterial genera ($\log_2$ scale); G-/G+ ratio, ratio of relative abundances of gramnegative to grampositive bacterial genera ($\log_2$ scale)

Supplementary Figure S4. Pairwise comparison of selected oral bacteriome characteristics on tumor surface and patient-matched healthy mucosa surface from patients with oral squamous cell carcinoma (N = 21).
