# Supplementary material for: Bacteriome-based oral dysbiosis index in patients with oral squamous cell carcinoma
Source: J Oral Microbiol. 2026 May 7;18(1):2668149. doi: 10.1080/20002297.2026.2668149 (PMC13159579; doi:10.1080/20002297.2026.2668149)
Supplement: Appendix_B_Supplementary_Methods_.docx [file ZJOM_A_2668149_SM6713.docx]

**Inclusion and exclusion criteria**

The study was conducted in compliance with STROBE (Strengthening the Reporting of Observational Studies in Epidemiology) guidelines. Patients with histopathological diagnosis of OSCC were recruited from the pool of patients attending the University Hospital Brno within the 2020-2022 according to the inclusion and exclusion criteria as presented in the flowchart (Figure 1). Only individuals with Czech or Slovak nationality above 18 years of age, with the legal capacity to manage their own affairs, who agreed with participation and signed an informed consent form after being fully informed about the project, its purposes, and their role in it, were included in the study. Patients with orthodontic appliances, toothless patients, as well as patients taking immunosuppressants, antibiotics in the last two months before the sample collection, and patients with acute respiratory infection in the last two weeks before the sample collection were excluded from the study. Additional exclusion criterium for patients was as follows: low quality of sequencing data (< 2000 reads per sample) from 16S rRNA amplicon sequencing.

**Clinical examination**

All patients were examined by an experienced maxillofacial surgeon from the Clinic of Maxillofacial Surgery by taking a complete medical history, performing clinical examination, and diagnostic imaging as needed to determine the staging of the OSCC – extent of the primary tumor, absence or presence and extent of regional lymph nodes, and the absence or presence of distant metastasis [1]. For the staging of the tumor, head and neck CT scans were taken for the evaluation of the tumor extent and nodal involvement. In addition, ultrasound examination of the abdomen as well as chest X-rays were performed for the assessment of distant metastases. Data about selected exposure factors, e.g., smoking, alcohol consumption, and oral hygiene was collected. Patients were categorized according to their smoking status as individuals who had never smoked (non-smokers), former smokers (ex-smokers), and active smokers (smoker) of cigarettes or other tobacco products. With respect to alcohol consumption, patients were categorized as teetotal, former drinker (ex-drinker), occasional drinker, and drinker (>2 alcohol beverages per day or three bottles of wine per week). Oral hygiene was assessed based on an inquiry and the examination of the dental status (missing teeth after the year 20 and the presence of tooth fillings) and the frequency of tooth-brushing according to the protocol by Liu et al. [2]. Additional clinical parameters such as tumor size, tumor location, perineural invasion, and bone invasion were also assessed.

**Sample collection**

Firstly, buccal swabs (Amies medium, Transystem^TM^, COPAN, Italy) from the healthy mucosa were taken for the identification of microscopic fungi. This sample was transported at 4°C for microbiological examination to the Department of Microbiology, St. Anne´s University Hospital.

For the bacteriome analysis, multiple oral swab samples were obtained in line with the previously published protocol [3] from the oral cavity of each participating patient during the initial examination using FLOQSwabs^®^ (COPAN, Italy). The preparation for sample collection required stringent standardization, with participants receiving clear instructions not to eat, chew gum, drink anything besides water, or perform any oral hygiene for 1 h before the procedure. Swabs were collected from the tumor surface, surface of the healthy buccal mucosa, healthy mucosa from the dorsum of the tongue, and from supragingival plaque. The swabs were immediately deposited in sterile Eppendorf tubes and stored at -80 °C until DNA isolation at RECETOX, Masaryk University.

Finally, a biopsy was taken from each tumor during surgical examinations, fixed in formalin, and embedded in paraffin (FFPE block) for subsequent conventional histopathological examination and determination of the p16 protein and selected oncogenic viruses at the Department of Pathology, University Hospital Brno.

**Histopathological examination**

Each FFPE sample was sectioned on a laminar flow table and placed in a tissue embedding cassette. Cassettes were placed in an automatic tissue processor (Milestone Logos, Italy), in which the samples were automatically treated with increasing ethanol concentrations, followed by xylene. The tissue was then embedded in paraffin on a tissue embedding unit (TES99). The parafﬁn blocks were serially cut into 3 µm sections for histopathological analysis. The sections were deparafﬁnized with sequences of xylene, hydrated in decreasing concentrations of ethanol, and washed with tap water before being kept in haematoxylin for 3 minutes. Sections were further washed in tap water for 10 minutes, immersed in eosin for 1 minute, and then in water for 5 minutes. After that, the sections were subjected to immersions in 70% ethanol, absolute ethanol, acetone-xylene solution, and xylene in an automatic staining unit (Leica ST5020). Finally, the specimen was mounted with a coverslip. Microscopic analysis of the haematoxylin-eosin-stained slides was performed by an experienced specialist trained in oral pathology using an optical microscope (Leica DM2000 LED, Germany). Besides, the confirmation of the diagnosis, histopathological grading, and p16 expression were determined from the biopsy.

**Analysis of p16 and selected oncogenic viruses**

For p16 staining, we used the CINtec® p16 Histology antibody (clone E6H4) (Ventana Medical Systems, USA). The protocol was based on heat-induced epitope retrieval (HIER) during CC1 conditioning (95 °C for 36 minutes), followed by incubation with the primary antibody (RTU) for 20 minutes. The detection kit used for both antibodies was UltraWiev DAB (Ventana Medical Systems, USA). Immunohistochemical staining (IHC) and *in situ* hybridization (ISH) were performed using the VENTANA BenchMark ULTRA system (Ventana Medical Systems, USA).

CMV IHC staining was performed using Anti-Cytomegalovirus antibody (clone CCH2+DDG9, RTU, Dako, USA). The staining protocol was based on heat-induced epitope retrieval (HIER) in pH9 buffer at 98 °C for 64 minutes, followed by incubation with the primary antibody for 52 minutes. ISH was performed using the INFORM EBER Probe (Ventana Medical Systems, USA) and the ISHiVIEWBlue Detection Kit (Ventana Medical Systems, USA) to detect cells expressing EBV-encoded RNA (EBER). The recommended staining protocol for the INFORM EBER Probe was used to perform the assay. EBER Positive Specimen Slides control material (Ventana Medical Systems, USA) was used for reference.

**Analysis of microscopic fungi**

The samples were resuspended in 1 ml of phosphate-buffered saline (PBS) and homogenized by vortexing. Subsequently, 100 mL of the resultant homogenate was inoculated onto Sabouraud's 4% maltose agar (Merck KGaA, Darmstadt, Germany) and CHROMagar Candida (CHROMagar, Paris, France). After 48 hours of incubation at 37 °C, yeast growth was assessed. All isolates were identified by Matrix-assisted laser desorption/ionization-time of flight (mass spectrometry (MALDI-TOF MS) using the MALDI BioTyper system (Bruker Daltonics, Switzerland) and FlexControl 3.4 software (Bruker Daltonics, Switzerland) according to the manufacturer's instructions. Mass spectra were processed using BioTyper 3.1 software (Bruker Daltonics, Switzerland). All samples were stored at 4°C and analyzed by MALDI-TOF mass spectrometry according to the manufacturer's instructions (Bruker). The analysis is described in detail in our previous study [4].

**Bacteriome analysis**

DNA was isolated from patient-matched swab samples using the DNA Mini Kit (Qiagen, Germany) in accordance with the manufacturer’s instructions for isolation of microbial DNA. In brief, 400 µl of ATL buffer and 20 µl of protein kinase K were added to the swab, the mixture was vortexed, and incubated for 80 minutes at 56 °C and 800 rpm (Thermo-Shaker TS-100C, Biosan, Latvia). Then, 5 µl of ribonuclease (RNase, 25 µg/ml) was added and incubated for 2 minutes at room temperature. To finish the lysis, 200 µl of the AL buffer was added, and the mixture was incubated for 10 minutes at 70 °C and 800 rpm (Thermo-Shaker TS-100C, Biosan, Latvia). Later, 200 µl of absolute ethanol was added. The liquid contents were transferred to the Spin Column and centrifuged for 1 minute at 6,000 × g. The extraction column was washed with 500 µl of AW1 buffer, centrifuged for 1 minute at 6,000 × g, and followed by the addition of 500 µl of AW2 buffer, centrifuged for 1 minute at 6,000 × g and 3 minutes at 16,000 × g to remove ethanol residues. Finally, 30 µl of AE buffer was pipetted on the column for DNA elution. DNA samples were stored at 4 °C, and after quality control using the Synergy HTX spectrophotometer (BioTek, USA) and electrophoresis on 1% agarose gel, the DNA samples were stored at -20 °C.

To amplify the hypervariable V3-V4 region of the gene for 16S rRNA, a polymerase Q5® High-Fidelity 2x Master Mix (New England BioLabs, USA) and specific Illumina primers (see Table A) were used. The PCR mixture (without the sample DNA) was decontaminated by adding 2 µl of 20x diluted 8-methoxypsoralen (8µg/µl, Sigma-Aldrich, USA), incubated for 90 minutes at 4 °C and for an additional 7 minutes under UV light. PCR was performed under these conditions: 30 s at 98 °C followed by 30 cycles (each cycle: 10 s at 98 °C followed by 15 s at 55 °C and then 30 s at 72 °C), finished by 2 min at 72 °C and then held at 4 °C; lid temperature was 105 °C. Then, PCR products were purified using SPRIselect beads (Beckman Coulter, USA), their concentration was measured in a fluorimeter Synergy HTX (BioTek, USA), and the samples were pooled equimolarly.

Libraries for subsequent next-generation sequencing were prepared according to the standard Illumina 16S metagenomic protocol with minor modifications at RECETOX, Masaryk University. For index PCR, Nextera XT indexes (Illumina, USA) were used. Sequencing was performed with a Miseq reagent kit V3 using MiSeq instrument according to the manufacturer's instructions (Illumina, USA) at CEITEC, Masaryk University.

**Table A.** Illumina primer sequences used for 16S rRNA amplification.

| Primer | Type of primer | Sequence |
| --- | --- | --- |
| IL2 | Forward | TCGTCGGCAGCGTCAGATGTGTATAAGAGACAGACGAAGTCCTACGGGNGGCWGCAG |
|  | Reverse | GTCTCGTGGGCTCGGAGATGTGTATAAGAGACAGAGCCACCGACTACHVGGGTATCTAATCC |
| IL4 | Forward | TCGTCGGCAGCGTCAGATGTGTATAAGAGACAGATAATGTCCTACGGGNGGCWGCAG |
|  | Reverse | GTCTCGTGGGCTCGGAGATGTGTATAAGAGACAGACGGCCCGACTACHVGGGTATCTAATCC |
| IL5 | Forward | TCGTCGGCAGCGTCAGATGTGTATAAGAGACAGAGGGTGTCCTACGGGNGGCWGCAG |
|  | Reverse | GTCTCGTGGGCTCGGAGATGTGTATAAGAGACAGACTGGCCGACTACHVGGGTATCTAATCC |
| IL6 | Forward | TCGTCGGCAGCGTCAGATGTGTATAAGAGACAGAGCCAGTCCTACGGGNGGCWGCAG |
|  | Reverse | GTCTCGTGGGCTCGGAGATGTGTATAAGAGACAGAAGGACCGACTACHVGGGTATCTAATCC |
| IL7 | Forward | TCGTCGGCAGCGTCAGATGTGTATAAGAGACAGAGTTCGTCCTACGGGNGGCWGCAG |
|  | Reverse | GTCTCGTGGGCTCGGAGATGTGTATAAGAGACAGACGCTCCGACTACHVGGGTATCTAATCC |
| IL8 | Forward | TCGTCGGCAGCGTCAGATGTGTATAAGAGACAGAATGCAGTCCTACGGGNGGCWGCAG |
|  | Reverse | GTCTCGTGGGCTCGGAGATGTGTATAAGAGACAGAATTAGCCGACTACHVGGGTATCTAATCC |
| IL9 | Forward | TCGTCGGCAGCGTCAGATGTGTATAAGAGACAGAATTTAGTCCTACGGGNGGCWGCAG |
|  | Reverse | GTCTCGTGGGCTCGGAGATGTGTATAAGAGACAGATCCTCCCGACTACHVGGGTATCTAATCC |
| IL11 | Forward | TCGTCGGCAGCGTCAGATGTGTATAAGAGACAGATCCTCGTCCTACGGGNGGCWGCAG |
|  | Reverse | GTCTCGTGGGCTCGGAGATGTGTATAAGAGACAGATCCCTCCGACTACHVGGGTATCTAATCC |
| IL13 | Forward | TCGTCGGCAGCGTCAGATGTGTATAAGAGACAGATCCCTGTCCTACGGGNGGCWGCAG |
|  | Reverse | GTCTCGTGGGCTCGGAGATGTGTATAAGAGACAGAGGCCTCCGACTACHVGGGTATCTAATCC |
| IL14 | Forward | TCGTCGGCAGCGTCAGATGTGTATAAGAGACAGACATTTGTCCTACGGGNGGCWGCAG |
|  | Reverse | GTCTCGTGGGCTCGGAGATGTGTATAAGAGACAGACCCAGCCGACTACHVGGGTATCTAATCC |
| IL15 | Forward | TCGTCGGCAGCGTCAGATGTGTATAAGAGACAGATTGCGTGTCCTACGGGNGGCWGCAG |
|  | Reverse | GTCTCGTGGGCTCGGAGATGTGTATAAGAGACAGACGGCTCCCGACTACHVGGGTATCTAATCC |
| IL16 | Forward | TCGTCGGCAGCGTCAGATGTGTATAAGAGACAGATAAAGAGTCCTACGGGNGGCWGCAG |
|  | Reverse | GTCTCGTGGGCTCGGAGATGTGTATAAGAGACAGACGACATCCGACTACHVGGGTATCTAATCC |

**Bioinformatic analysis**

Bioinformatic analysis of sequencing data was described in detail previously [5]. Our analysis was initiated by demultiplexing raw fastq files utilizing Cutadapt v.3.4 for further processing; we employed the nf-core/ampliseq pipeline v.2.6.1. After demultiplexing, the overall quality was assessed using FastQC v.0.11.9. The reads were trimmed to a minimal Phred score of 25 and lengths of 292 bp for forward reads and 205 bp for reverse reads. Reads with mean Phred scores of less than 25 were filtered out. Reads underwent processing using the DADA2 (Divisive Amplicon Denoising Algorithm) pipeline as a part of the nf-core/ampliseq. DADA2 1.22.0 was applied. From the merged sequences, amplicon sequence variants (ASVs) were inferred using DADA2. We used Barrnap v.0.9 for the taxonomic prediction of the kingdom of the rRNA origin to filter out Archaea, mitochondria, and Eukaryotes. Taxonomic classification was performed using the DADA2 classifier (RDP naive Bayesian classifier to the SILVA 138.1 reference database). QIIME 2 v.2022.11.1 was used for the generation of absolute and relative feature/taxa count tables and the generation of basic taxa abundance plots. Additionally, alpha and beta diversity indices were computed using QIIME2. Functional profiling of sequences was conducted in Picrust2 v.2.5.0 as a part of nf-core/ampliseq.

**Statistical analysis**

All statistical analyses were conducted in **R (version 4.1.2)** [6]. A significance threshold of α = 0.05 was applied. Where appropriate, *p*-values were adjusted for multiple testing using the Benjamini–Hochberg procedure.

Samples with fewer than 2,000 sequencing reads were excluded from the dataset. Subsequently, patients without a complete set of samples from all four oral sites (tumor surface, buccal mucosa, tongue, and supragingival dental plaque) were excluded from the final statistical evaluation.

Due to the sparse and non-normal nature of bacteriome data, non-parametric statistical methods were employed. Paired tests were used when applicable, specifically the Wilcoxon signed-rank test for comparisons between two groups and Friedman’s test for repeated measures involving more than two groups. For categorical variables, Fisher’s exact test was applied. Test results, along with summary statistics, were compiled into tables generated using the **gt** package in R [7].

For subsequent bacteriome analyses, alpha diversity indices and Bray-Curtis dissimilarity matrix were calculated using the **vegan** package in R [8]. Principal component analysis (PCA) was performed on central log ratio (CLR) transformed data and visualized with PCA tools package [8].

Alpha diversity indices and compositional ratios (anaerobes/aerobes, G-/G+, and bbODI) were visualized on a log₂ scale to improve interpretability and accommodate skewed distributions. Group comparisons were displayed using violin plots created with the **ggplot2** package [9].

To investigate associations between clinical parameters as well as bacteriome characteristics (indices and relative abundances of the top 25 bacterial genera) on tumor surface and patient-matched healthy mucosa samples, a correlation matrix was computed and then visualized using the **corrplot** package [10]. Depending on the variable type, different correlation measures were applied: Cramér’s V for categorical variables, rank-biserial correlation for binary versus continuous variables, and Spearman’s rank correlation for associations between continuous variables. Statistical significance of correlations was assessed using the corresponding tests.

Further visualization of the bacteriome composition and sample-level variation was performed using heatmaps generated with the **ComplexHeatmap** [11] and **circlize** [12] packages. A custom visualization was developed to highlight compositional differences across sample matrices.

**References**

[1] Brierley JD, Gospodarowicz MK, Wittekind C. TNM Classification of Malignant Tumours. John Wiley & Sons; 2017.

[2] Liu Q-Y, Liao Y, Wu Y-X, et al. The Oral Microbiome as Mediator between Oral Hygiene and Its Impact on Nasopharyngeal Carcinoma. Microorganisms 2023;11:719. <https://doi.org/10.3390/microorganisms11030719>.

[3] Marincak Vrankova Z, Brenerova P, Bodokyova L, et al. Tongue microbiota in relation to the breathing preference in children undergoing orthodontic treatment. BMC Oral Health 2024;24:1259. <https://doi.org/10.1186/s12903-024-05062-3>.

[4] Cvanova M, Ruzicka F, Kukletova M, et al. Candida species and selected behavioral factors co-associated with severe early childhood caries: Case-control study. Front Cell Infect Microbiol 2022;12:943480. <https://doi.org/10.3389/fcimb.2022.943480>.

[5] Szaraz D, Bohm J, Cerulova S, et al. Bacterial genera in the fluids from apical periodontitis-related radicular cysts: An observational study. Int Endod J 2025;58:902–15. <https://doi.org/10.1111/iej.14220>.

[6] R Core Team. R: a language and environment for statistical computing. Vienna, Austria: R Foundation for statistical Computing. <https://www.r-project.org/>, 2021 (accessed August 21, 2025).

[7] Iannone R, Cheng J, Schloerke B, al. gt: Easily Create Presentation-Ready Display Tables. R package version 1.0.0. [https://CRAN.R-project.org/package=gt](https://cran.r-project.org/package=gt), 2025 (accessed 21 August 2025).

[8] Oksanen J, Simpson GL, Blanchet FG, et al. vegan: Community Ecology Package. R package version 2.6-10. [https://CRAN.R-project.org/package=vegan](https://cran.r-project.org/package=vegan), 2025 (accessed 21 August 2025).

[9] Wickham H. ggplot2: Elegant Graphics for Data Analysis. Springer International Publishing; 2016.

[10] Taiyun W, Viliam S. R package “corrplot”: Visualization of a Correlation Matrix (Version 0.95). <https://github.com/taiyun/corrplot>, 2025 (accessed 21 August 2025).

[11] Gu Z, Eils R, Schlesner M. Complex heatmaps reveal patterns and correlations in multidimensional genomic data. Bioinformatics 2016;32:2847–9. <https://doi.org/10.1093/bioinformatics/btw313>.

[12] Gu Z, Gu L, Eils R, et al. circlize implements and enhances circular visualization in R. Bioinformatics 2014;30:2811–2. <https://doi.org/10.1093/bioinformatics/btu393>.
