# Supplementary material for: Bacteriome-based oral dysbiosis index in patients with oral squamous cell carcinoma
Source: J Oral Microbiol. 2026 May 7;18(1):2668149. doi: 10.1080/20002297.2026.2668149 (PMC13159579; doi:10.1080/20002297.2026.2668149)
Supplement: Appendix C_Supplementary Table 1.docx [file ZJOM_A_2668149_SM6711.docx]

**Supplementary Table 1.** Overview of oral dysbiosis indices in adults

| **Index** | **Disease** | **Samples** | **Components** | **Method** | **Study design** | **Population** | **Reference** |
| --- | --- | --- | --- | --- | --- | --- | --- |
| bbODI | OSCC | Swabs from oral mucosa | the sum of the relative abundances of the genera from the orange and red complex bacteria (*Fusobacterium, Prevotella, Parvimonas*, *Peptostreptococcus*, *Porphyromonas, Tannerella*, and *Treponema*) divided by the sum of the relative abundances of commensal genera (*Streptococcus, Veillonella, Rothia, Gemella, Actinomyces*, *Granulicatella*, and *Haemophilus*) | 16S rRNA amplicon sequencing | Case series with paired samples (tumor vs. healthy mucosa) | Czech and Slovak | This study |
| SMDI | Periodontitis | Subgingival plaque and saliva | Relative abundance of genera *Fretibacterium, Treponema, Actinomyces* | 16S rRNA amplicon sequencing | Case-control (periodontally healthy and periodontitis-affected individuals) | Mixed (USA, UK, Sweden, South Korea) | Chen et al., 2021  Chew et al., 2025 |
| RAS-associated dysbiosis index | RAS | Mucosa sampling using polyvinylidene difluorid membrane and saliva | Decreased *Streptococcus salivarius* and increased *Acinetobacter johnsonii* | Pyrosequencing of the gene for 16S rRNA | Case-control (RAS patients with active lesions and healthy controls) | South Korea | Kim et al. 2016 |
| Microbiome biomarker panel to predict OSCC and OPSCC | OSCC and OPSCC | Oral rinse | Relative abundances of genera *Rothia, Haemophilus, Corynebacterium, Paludibacter, Porphyromonas, Oribacterium*, and *Capnocytophaga* | 16S rRNA amplicon sequencing | Case-control (Patients with OSCC and OPSCC, high-risk individuals, healthy control) | Australian | Lim et al. 2018 |
| MDI | OSCC and OPSCC | FFPE (tumor tissue) | Ratio of the total abundances of genera *Shlegelella* and *Methyloversatilis* and the total abundances of genear *Bacillus*, *Lactobacillus* and *Sphingomonas* | 16S rRNA amplicon sequencing | Case series (associations with OS and DFS) | Chinese | Dou et al. 2022 |
| MDI | OSCC | Saliva | log10 of the total abundance of *Streptococcus*, *Capnocytophaga*, and *Gemella* divided by the total abundance of *Megasphaera*, *Lachnospiraceae_[G-2]*, *Prevotella*, *Stomatobaculum*, *Veillonella*, *Lachnoanaeroba-culum*, *Peptostreptococcaceae_[XI][G-1]* | 16S rRNA amplicon sequencing | Case-control (Patients with OSCC and healthy controls) | Chinese | He et al. 2024 |

bbODI, bacteriome-based oral dysbiosis index; SMDI, subgingival microbial dysbiosis index; MDI, microbial dysbiosis index; RAS, recurrent aphthous stomatitis; OSCC, oral squamous cell carcinoma; OPSCC, oropharyngeal squamous cell carcinoma; FFPE, formalin-fixed paraffin-embedded; OS, overall survival; DFS, disease free survival

**References**

Chew RJJ, Tan KS, Chen T, et al. Quantifying periodontitis-associated oral dysbiosis in tongue and saliva microbiomes-An integrated data analysis. J Periodontol 2025;96:55–66. https://doi.org/10.1002/JPER.24-0120.

Chen T, Marsh PD, Al-Hebshi NN. SMDI: An Index for Measuring Subgingival Microbial Dysbiosis. J Dent Res 2022;101:331–8. <https://doi.org/10.1177/00220345211035775>.

Kim Y-J, Choi YS, Baek KJ, et al. Mucosal and salivary microbiota associated with recurrent aphthous stomatitis. BMC Microbiol 2016;16 Suppl 1:57. <https://doi.org/10.1186/s12866-016-0673-z>.

Lim Y, Fukuma N, Totsika M, et al. The Performance of an Oral Microbiome Biomarker Panel in Predicting Oral Cavity and Oropharyngeal Cancers. Front Cell Infect Microbiol 2018;8:267. <https://doi.org/10.3389/fcimb.2018.00267>.

Dou Y, Ma C, Wang K, et al. Dysbiotic tumor microbiota associates with head and neck squamous cell carcinoma outcomes. Oral Oncol 2022;124:105657. <https://doi.org/10.1016/j.oraloncology.2021.105657>.

He B, Cao Y, Zhuang Z, et al. The potential value of oral microbial signatures for prediction of oral squamous cell carcinoma based on machine learning algorithms. Head Neck 2024;46:1660–70. https://doi.org/10.1002/hed.27795.
